# Association between Changing Mortality of Digestive Tract Cancers and Water Pollution: A Case Study in the Huai River Basin, China

**DOI:** 10.3390/ijerph120100214

**Published:** 2014-12-23

**Authors:** Hongyan Ren, Xia Wan, Fei Yang, Xiaoming Shi, Jianwei Xu, Dafang Zhuang, Gonghuan Yang

**Affiliations:** 1 State Key Laboratory of Resources and Environmental Information System, Institute of Geographic Sciences and Natural Resources Research, Chinese Academy of Sciences, Beijing 100101, China; E-Mails: renhy@igsnrr.ac.cn (H.R.); yangfei@igsnrr.ac.cn (F.Y.); 2 Institute of Basic Medical Sciences, Chinese Academy of Medical Sciences, 5 Dong Dan San Tiao, Beijing 100005, China; E-Mail: wanxiasnake@163.com; 3 Chinese Center for Disease Control and Prevention, Beijing 102206, China; E-Mails: sxmcdc@163.com (X.S.); xjw1214@163.com (J.X.)

**Keywords:** increasing mortality, digestive tract cancers, water pollution, drinking water safety, Huai River Basin

## Abstract

The relationship between the ever-increasing cancer mortality and water pollution is an important public concern in China. This study aimed to explore the association between serious water pollution and increasing digestive cancer mortality in the Huai River Basin (HRB) in China. A series of frequency of serious pollution (FSP) indices including water quality grade (FSP_WQG_), biochemical oxygen demand (FSP_BOD_), chemical oxygen demand (FSP_COD_), and ammonia nitrogen (FSP_AN_) were used to characterize the surface water quality between 1997 and 2006. Data on the county-level changing mortality (CM) due to digestive tract cancers between 1975 and 2006 were collected for 14 counties in the study area. Most of investigated counties (eight) with high FSP_WQG_ (>50%) distributed in the northern region of the HRB and had larger CMs of digestive tract cancers. In addition to their similar spatial distribution, significant correlations between FSP indices and CMs were observed by controlling for drinking water safety (DWS), gross domestic product (GDP), and population (POP). Furthermore, the above-mentioned partial correlations were clearly increased when only controlling for GDP and POP. Our study indicated that county-level variations of digestive cancer mortality are remarkably associated with water pollution, and suggested that continuous measures for improving surface water quality and DWS and hygienic interventions should be effectively implemented by local governments.

## 1. Introduction

Water pollution has been a common environmental problem as well as an important public health concern in China [1,2]. Among the seven main river basins in China, the Huai River Basin (HRB) is the most worrisome because of its dense population, intensive water network, and long-term serious water pollution [3]. Recently, spatiotemporal variations of water pollution in the HRB have been well characterized, indicating that the heavily polluted regions were mostly distributed in the area along several tributaries of the Ying, Guo, and New Sui Rivers, as well as the area north of Nansi Lake [4,5], which was mainly due to dramatic population growth, the rapid development of industrial and agricultural production, and the increase of township enterprises since 1990s [6].

Since 2000, the deteriorated public health status is another public concern in the HRB [5]. Retrospective cause of death survey data for China in the 1970s show that there were low death rates from cancer in the upper and middle reaches of the Huai River at that time. Meanwhile, the mortality rate for digestive system cancers, with the exception of esophageal cancer, was lower than the national average [7]. Our earlier investigation during 2004–2006 found that some counties in the HRB have suffered from increasing incidence and mortality rates for malignant tumors [8,9]. In particular, some villages (*i.e.*, “cancer villages”) in the HRB possessing high mortality from cancers have been widely reported by the domestic media since 2004 [9,10], and this has attracted the attention of various parties to the issue of the high rates of cancer in the area. The public is eager to know whether the occurrence of “cancer villages” was directly related to water pollution or not. However, there is little evidence demonstrating the relationship between the ever-growing cancer mortality and surface water pollution.

In recent years, the causal relationship between water environment and cancer epidemiology has been extensively studied on multilevel scales [11,12,13,14,15]. The populations in areas with a high exposure to organic or inorganic pollutants in water environments may suffer from high cancer or non-cancer mortality [16,17,18,19,20,21,22]. These investigations are useful for understanding of both the increasing mortality of these malignant tumors in the HRB and the effects of water pollution on the distribution and severity of digestive tract cancers. However, most of the previous investigations were not quantitative but rather were qualitative analyses based on static cancer mortality data and environmental factors.

In this study, we aimed to: (1) to identify the spatial variations in the changing mortality (CM) of digestive tract cancers during 1975–2006 at the county level, and (2) to quantitatively explore the relationship between CMs to the spatiotemporal features of surface water pollution in the HRB. The results of this study may be useful for addressing public concerns regarding the health effects of water pollution and may be valuable for local governments as a tool for implementing effective measures.

## 2. Results

### 2.1. County-Level Water Pollution

**Figure 1:**
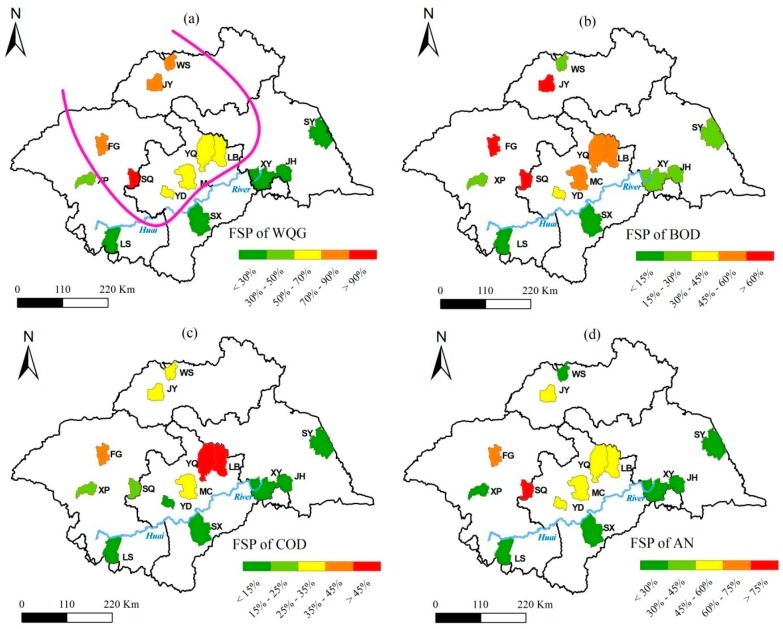
Spatiotemporal variations of water pollution in terms of county-level FSP indices ((**a**) FSP_WQG_, (**b**) FSP_BOD_, (**c**) FSP_COD_, and (**d**) FSP_AN_) during 1997–2006.

In terms of FSP_WQG_, as illustrated in Figure 1a, the 14 counties were divided into two groups (outlined by a purple separator curve). JY, WS, FG, SQ, YD, MC, YQ, and LB County, located in the northern area of the HRB, belonged to the first group because of their higher FSP_WQG_ (above 50%). In the second group (FSP_WQG_ < 50%) with the exception of XP County, five counties (LS, SX, JH, XY, and SY County) were distributed along the southern side of the Huai River.

In the first group, SQ and YD County reported FSP_COD_ of less than 25% and 15%, respectively (Figure 1c). Similarly, WS had a FSP_BOD_ of less than 30% (Figure 1b). However, these three counties simultaneously experienced serious water pollution caused by BOD (SQ and YD), COD (WS), and AN (SQ and YD), as shown in Figure 1b–d. These results indicated that most of investigated counties in the northern part of the central HRB suffered from serious surface water pollution caused by excessive concentrations of BOD, AN, and COD.

### 2.2. CMs of Digestive Tract Cancers

**Figure 2:**
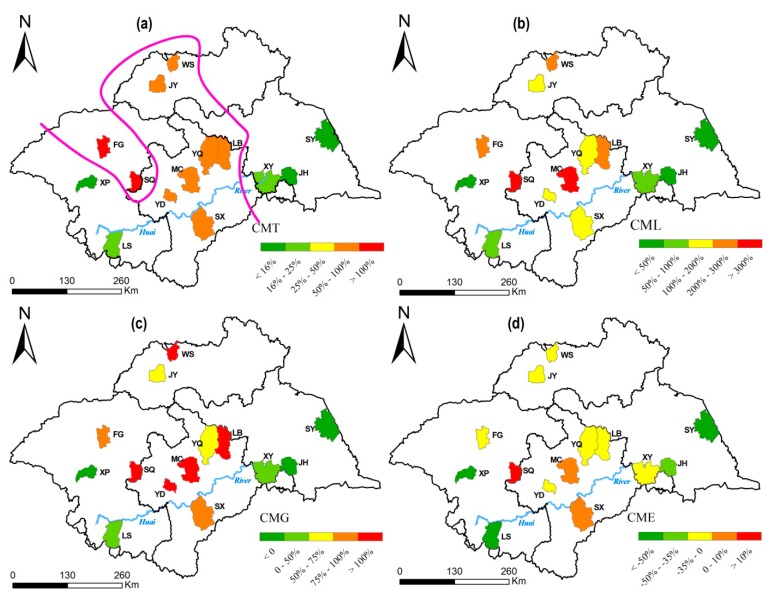
Spatial distribution of CMT (**a**), CML (**b**), CMG (**c**), and CME (**d**) of 14 counties from 1975 to 2006.

Based on the county-level CMs as illustrated in Figure 2a–c, the 14 counties were divided into three groups (outlined by a purple separator curve in Figure 2a): XP-LS-XY-JH-SY (Group I), YD-SX-MC-YQ-LB-WS-JY (Group II), and FG-SQ (Group III). The counties in Group II and Group III had relatively higher CMs (CMT > 0.25 times, CML > 1.0 times, and CMG > 0.5 times) and were mostly distributed in the central region across the HRB from the south to the north. Moreover, the esophageal cancer mortality from SQ, MC, and SX displayed an upward trend (CME > 0), whereas the remaining 11 counties’ esophageal cancer mortality rates tended to decline (CME < 0) between 1975 and 2006 (Figure 2d). The county-level CMs presented similar spatial distributions to those of the FSP indices as a whole.

### 2.3. Relation of Water Pollution to CMs at the County Level

In addition to similar spatial distribution, the county-level CMs tended to be quantitatively related to FSP indices as shown in Table 1. Except for FSP_COD_, the FSP indices were closely correlated with the CMs at the county level according to their linear correlations. The county-level CMT, CML, and CMG were notably related to FSP_WQG_ and FSP_AN _at a significance level of 0.01. In particular, FSP_AN_ presented a stronger correlation with CMT (r = 0.83, *P* < 0.01) and CME (r = 0.57, *P* < 0.05) and had a similar association with CML (r = 0.72, *P* < 0.01) and CMG (r = 0.71, *P* < 0.01). The counties with higher FSP indices (FSP_AN_, FSP_WQG_, and FSP_BOD_) are experiencing increasing rates of digestive cancer mortality.

**Table 1:** Linear correlation between CMs and the selected factors at the county scale.

CMs	FSP_WQG_	FSP_BOD_	FSP_COD_	FSP_AN_	GDP	POP	DWS
CMT	0.78 ^‡^	0.72 ^‡^	0.42	0.83 ^‡^	−0.13	0.45	−0.58 ^†^
CML	0.72 ^‡^	0.62 ^†^	0.37	0.72 ^‡^	−0.07	0.39	−0.54 ^†^
CMG	0.73 ^‡^	0.62 ^†^	0.32	0.71 ^‡^	−0.11	0.35	−0.45
CME	0.47 *	0.4	0.17	0.57 ^†^	−0.34	0.32	−0.37

Note: *, ^†^, and ^‡^ denote a significance levels of 0.10, 0.05, and 0.01, respectively (two-tailed).

Among the rest three factors (GDP, POP, and DWS), only DWS was significantly related to both CMT (r = −0.58, *P* < 0.05) and CML (r = −0.54, *P* < 0.05). In addition, the correlation between them was decreased to varying degrees when DWS-GDP-POP or GDP-POP were considered as control variables (Table 2). If controlling for DWS-GDP-POP, both FSP_AN_ and FSP_WQG_ were closely associated with CMT, CML, and CMG. In comparison, their relationships were enhanced in case of not controlling for DWS (i.e. controlling for GDP-POP). In this case, FSP_BOD_ was also significantly correlated with CMT (r = 0.62, *P* < 0.05), CML(r = 0.50, *P* < 0.10), and CMG (r = 0.50, *P* < 0.10). These results showed that the association between FSP indices and CMs was obviously affected by DWS, GDP, and POP.

**Table 2:** Partial correlation between CMs and FSP indices with various control variables.

CMs	Control Variables: GDP-POP-DWS					Control Variables: GDP-POP			
	FSP_WQG_	FSP_BOD_	FSP_COD_	FSP_AN_		FSP_WQG_	FSP_BOD_	FSP_COD_	FSP_AN_
CMT	0.67 ^†^	0.54	0.08	0.70 ^†^		0.72 ^‡^	0.62 ^†^	0.21	0.73 ^‡^
CML	0.58 *	0.41	0.05	0.57 *		0.65 ^†^	0.50 *	0.18	0.63^ †^
CMG	0.66 ^†^	0.45	0.06	0.56 *		0.67^ †^	0.50 *	0.15	0.60 ^†^
CME	0.27	0.07	−0.14	0.22		0.29	0.12	−0.08	0.24

Note: *, ^†^, and ^‡^ denote a significance levels of 0.10, 0.05, and 0.01, respectively (two-tailed).

## 3. Discussion

In this study, we found that the spatial distribution of county-level CMs tended to be similar to that of FSP indices, and that 14 counties’ CMs were significantly correlated with FSP indices in the HRB. Meanwhile, lower DWS clearly contributed to the increasing mortality of digestive cancer in the counties with serious water pollution. This study would be a useful reference for epidemiologists to conduct detailed investigations on public health concerns related to eco-environmental changes. Moreover, our findings could be valuable for local governments when making decisions on environmental management efforts and improving the condition of drinking water.

A reasonable evaluation of the effects of water pollution on public health depends on an analysis of the variations in water quality. Compared with traditional statistical methods aimed at analyzing temporal trends of water quality [23,24,25], the spatial interpolation employed in this study was more useful for obtaining spatiotemporal variations in water quality [4]. Thus, the county-scale data of water pollution data were easily utilized for evaluating the relationship between increasing cancer mortality and serious water pollution. Furthermore, the application of the CMs of digestive cancers in this study supplied more powerful evidence than the static cancer mortality data employed in previous studies. Accordingly, our results are useful for addressing public concerns regarding the relation of the ever-increasing mortality of digestive system cancers (*i.e.*, total digestive tract, liver, and gastric cancer) to long-term and large-scale serious water pollution in some regions of the HRB.

In previous studies, it has been proved that serious water pollution in the HRB was mainly caused by agricultural fertilization and pesticide spraying, livestock and poultry production, small-scale township enterprises, and domestic pollution [4,6]. In polluted water, chemical contaminants, including microcystins (MC), nitrate, nitrite, heavy metal, and N-nitroso compounds (NOC), had been detected to varying degrees [1,2,3,22,26,27,28], and had been identified as common carcinogens or promoters of digestive system cancers [13,26,28,29,30,31,32,33]. Moreover, Wan,* et al.* [9] have demonstrated that both liver cancer and gastric cancer are related to water pollution in some small areas of the HRB. These findings could validate the significant correlation between CMs (CMT, CML, and CMG) and FSP indices in this macroscopic investigation. Digestive system cancers are also closely related to other risk factors apart from the above-mentioned chemical pollutants [15,34,35,36]. However, the attribution of these CMs to water pollution should be further investigated by comparing water pollution with other risk factors. In this way, the relationship between CME and water pollution might be well explored for accommodating their weak association in this study. In addition, some probably unknown risk factors may be revealed by further microscopic researches, which could also be helpful for exploring these unknown factors’ implications for esophageal cancer, especially in the HRB. Even so, we believe that serious water pollution has played an important role in deteriorating public health status in the HRB.

The geographic information system (GIS) method has been widely employed to characterize the spatial patterns of cancers or carcinogens [12,36,37]. Through spatial overlay mapping, Yu and Zhang [10] inferred that 94 percent of all the “cancer villages” in China were related to regional water pollution in the past decades. However, in contrast with these qualitative investigations, our study successfully emphasized spatial coherency of cancer mortality variations and water pollution in terms of their significant correlations (Table 1 and Table 2). Meanwhile, spatial coherency and positive correlation between county-level CMs and FSP indices tended to be more similar and significant when we deleted SX County from the dataset (Supplementary Information Table S1 and Table S2). Accordingly, we cautiously speculate that there were probably unknown factors apart from water pollution in this county. Nevertheless, our results indicated that increased mortality of digestive tract cancers in the HRB is spatially and quantitatively related to water pollution.

In addition, much attention should be paid to DWS, although the relationships between DWS and CMs were relatively weak. To our knowledge, the average DWS (mean value, 13.7%) of these 14 counties was much lower than the average value of all the counties in the HRB (28.9%) and the national level (55.1%). When only GDP and POP were considered as control variables, correlations between CMs and FSP indices were stronger than those in case of controlling for DWS, GDP, and POP. These results implied that lower DWS tended to be a potential risk factor for increasing digestive cancer mortality, especially in the counties with larger FSP indices.

A few limitations of this study warrant mention. First, our investigation, which was based on 14 counties, requires further validation for the remaining counties (170 or so) in the HRB although current quantitative results preliminarily satisfied the spatial and quantitative correlations between increasing cancer mortality rates and water pollution. Additionally, the late establishment of projects on that monitored water quality in the 1980s led to a deficiency of surveillance data in the 1970s, which restrained the comparison analysis of water quality between the 1970s and 2004–2006. Finally, the capability of FSP indices should be improved to reflect more contaminants, such as heavy metals, so that the association between water pollution and digestive tract cancer mortality could be better explored.

## 4. Materials and Methods

**Figure 3:**
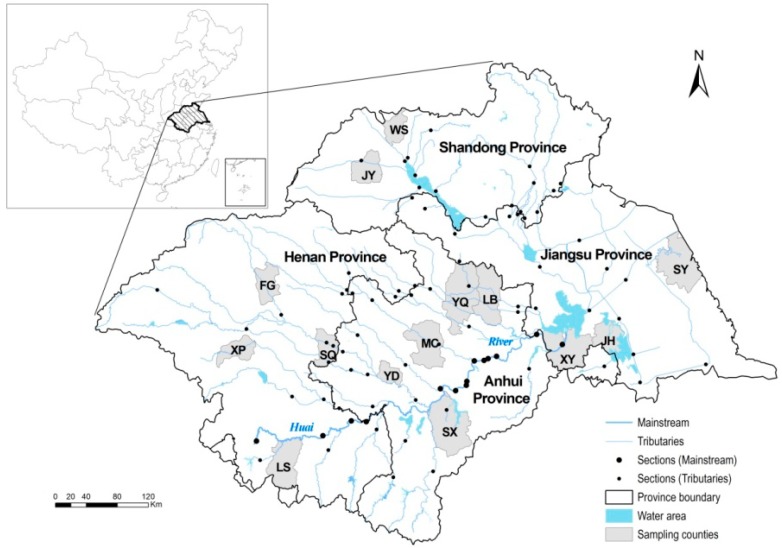
Illustration of 14 counties in the HRB and water quality monitoring sections of the Huai River System.

As illustrated in Figure 3, 14 counties, including Wenshang (WS), Juye (JY), Fugou (FG), Xiping (XP), Shenqiu (SQ), Luoshan (LS), Yingdong (YD), Mengcheng (MC), Yongqiao (YQ), Lingbi (LB), Shouxian (SX), Sheyang (SY), Xuyi (XY), and Jinhu (JH) County, are evenly located along the tributaries in the HRB, which is the most densely inhabited river basin and the main agricultural area of China.

In this study, two main regions, including the southern and northern part, were divided by the mainstream of the Huai River.

### 4.1. Data Collection

#### 4.1.1. Surface Water Quality

In view of the hysteresis effects (about 10–15 years) of environmental pollution on public health [38] as well as data availability, our study proposed to select water environment monitoring data from a period approximately 10 years earlier (*i.e.*, since 1990s) than the health data (in 2004–2006). In total, data from 86 state-controlled sections of the Huai River System, including 14 mainstream-sections and 72 tributary-sections as illustrated in Figure 3, were extracted from the series of China Environmental Quality Reports [3], which were published by the State Environmental Protection Administration from 1998 to 2007. In this dataset, three important indicators of water quality, including biochemical oxygen demand (BOD), chemical oxygen demand (COD), and ammonia nitrogen (AN), were consecutively collected; the comprehensive indicator termed water quality grade (WQG) was also collected.

#### 4.1.2. Cancer Mortality Data

As mentioned above, water quality data in 1997–2006 were selected and would be linked to the health data in 2004–2006. According to the objectives of this study, mortality data of digestive system cancers in the middle 1990s would be considered as the baseline for calculating CMs of digestive tract cancers. But we failed to collect this regular baseline data, and then acquired cancer mortality data in the middle 1970s. To our knowledge, HRB was always a less developed region with predominantly conventional agricultural production (*i.e.*, less environmental pollution) till the late 1980s. Therefore, it was assumed in this study that mortality rates of digestive system cancers were basically unchanged between the middle 1990s and 1970s.

The cancer mortality dataset of the 14 counties was extracted from the first nationwide epidemiological survey on cause of death conducted in 1973–1975 [39] and from a three-year retrospective survey on cause of death launched in these counties during 2004–2006 [8].

From this dataset, digestive tract cancers, including total digestive, liver, gastric, and esophageal cancers were selected for this study, according to their correspondent codes of International Standards for Disease Classification (ICD-10) as shown in Table 3.

**Table 3:** ICD codes of some causes of death from cancer.

Deaths	ICD-10
Neoplasm	C00-D48
Digestive cancer	C15-C20
Esophagus cancer	C15
Gastric cancer	C16
Liver cancer	C22

### 4.2. Socioeconomic Factors

The demographic data were based on the village-household population reported by these 14 counties during 2004–2006. To check the reliability of the age and gender distributions, this dataset was cross-referenced against census data in 2000 (published by National Bureau of Statistics of China and accessed via http://www.stats.gov.cn/tjsj/ndsj/renkoupucha). The county population (POP) is the sum of the individual village populations.

Together with the POP, the county-level gross domestic product (GDP) in 2005 and drinking water safety (DWS) in 2005 were also considered as control variables for evaluating the association between water pollution and CMs. GDP could reflect regional socioeconomic levels and people’s living standard. Similarly, DWS denoted the proportion of the population that was covered by the centralized water supply in 2005. County-level GDP and DWS data were supplied by local statistic bureaus.

### 4.3. Indices Calculation

#### 4.3.1. Frequency of Serious Pollution

The frequency of serious pollution (FSP) was employed to represent the spatiotemporal features of water pollution for the HRB and for each county. First, the FSP indices of WQG (FSP_WQG_), BOD (FSP_BOD_), COD (FSP_COD_), and AN (FSP_AN_), were respectively acquired by the following formula, and then were considered to be temporal changes of water pollution for each state-controlled section:

$${\mathrm{FSP}}_{\mathrm{Indicator}}=Y_{p}/Y_{t}$$

where Y*_p_* is the occurrence (years) of water quality indicators of grade V (*i.e.*, mainly suitable for agricultural uses and general scenic purposes) and grade V+ (*i.e.*, one of water quality indicators exceeding Grade V’s requirements) defined by the National Standard [40]; Y*_t_* is the total observation time (years) of water quality.

Secondly, the spatial interpolation (inverse distance weighted) method was applied to conduct the spatialization FSP indices of 86 sections with the resolution of 1 × 1 km^2^, which was described in detail in our earlier study [4]. Accordingly, the spatial distribution of FSP_WQG_, FSP_BOD_, FSP_COD_, and FSP_AN_ were subsequently obtained for the entire basin.

In the next step, the mean FSP values of these 14 counties (polygons) were acquired in Arcgis10.0 (ESRI, Redlands, CA, USA) by using the zonal statistics function of the spatial analysis tools, and then used to represent the mean frequency or possibility of serious pollution of water quality at the county level.

#### 4.3.2. CM of Digestive Tract Cancers

Using the cause of death codes (Table 3) of cause of death, the population that died of cancer, and the demographic data (including reported population data and population census data in 2000 accessed via http://www.stats.gov.cn/tjsj/ndsj/renkoupucha), we obtained some analysis indices including the annual mean population, crude mortality rates, standardized cancer mortality rates, and CM indices for the total digestive tract (CMT), liver cancer (CML), gastric cancer (CMG), and esophageal cancer (CME) using the equations below:

$$\bar{P}=(P_{l}+P_{t}\times 2+P_{n})/4$$

where $\bar{P}$ is the annual mean population; $P_{l}$, $P_{t}$, and $P_{n}$ represent the population at the end of the last, present, and next year, respectively:

$$p_{i}=(P_{d}/\bar{P})\times 100\%$$

where $p_{i}$ is the crude mortality rate; $P_{d}$ is the population that died of cancer:

$$P\prime =\sum^{\text{​}}(\frac{{Ν}_{i}}{Ν})\times p_{i}$$

where P′ is the standardized cancer mortality rate; $N_{i}$ and N. denote the age population and the total population of the census data in 2000, respectively. In this way, the mortality rates of different counties and different years are comparable:

$${\mathrm{CM}}_{i}=(P_{i,2004-06}^{\text{′}}-P_{i,1973-75}^{\text{′}})/P_{i,1973-75}^{\text{′}}$$

where $P_{i,2004-06}^{\text{′}}$ and $P_{i,1973-75}^{\text{′}}$ are the standardized mortality rates of total digestive, liver, gastric, and esophageal cancers in 2004–2006 and 1973–1975. Accordingly, ${\mathrm{CM}}_{i}$ represents CMT, CML, CMG, and CME.

### 4.4. Correlation Analysis

To evaluate the county-level association between CMs and selected factors (FSP indices, POP, GDP, and DWS), correlation analysis, including simple linear and partial correlations, was conducted using SAS9.0 software (SAS Institute Inc., Cary, NC, USA).

## 5. Conclusions

In summary, the increasing mortality rates of digestive tract cancers were spatially and quantitatively correlated with surface water pollution at the county level in the HRB with low DWS. This study would be evaluable for epidemiologists to explore the potential association between the change in cancer mortality and environmental pollution in some regions with similar surface water pollution in the past decades. In addition, we suggest that continuous measures for improving surface water quality, DWS and hygienic interventions should be effectively implemented by local governments in the HRB.

## Supplementary Files

Supplementary File 1

## References

[1] Kan H. (2009). Environment and health in China: Challenges and opportunities. Environ. Health Perspect..

[2] Wu C., Maurer C., Wang Y., Xue S., Davis D.L. (1999). Water pollution and human health in China. Environ. Health Perspect..

[3] Ministry of Environmental Protection of the People’s Republic of China (2008). China Environmental Quality Report 1998–2007.

[4] Ji W., Zhuang D., Ren H., Jiang D., Huang Y., Xu X., Chen W., Jiang X. (2013). Spatiotemporal variation of surface water quality for decades: A case study of huai river system, China. Water Sci. Technol..

[5] Yang G.H., Zhuang D.F. (2014). Atlas of the Water Environment and Digestive Cancer Mortality in the Huai River Basin.

[6] Zhang Y., Xia J., Liang T., Shao Q. (2010). Impact of water projects on river flow regimes and water quality in Huai River Basin. Water Resour. Manage..

[7] Editorial Committee for the Atlas of Cancer Mortality in the People’s Republic of China (1979). Atlas of Cancer Mortality in the People’s Republic of China.

[8] China CDC (2006). Study Report on Key Areas of Cancer Incidence in Huai River Basin and Risk Factors (Restricted).

[9] Wan X., Zhou M., Tao Z., Ding D., Yang G. (2011). Epidemiologic application of verbal autopsy to investigate the high occurrence of cancer along the Huai River Basin, China. Popul. Health Metrics.

[10] Yu J.L., Zhang S.Q. Analysis on Environmental Pollution and Public Health Reflected by “Cancer Villages” in China. Proceedings of the Annual Meeting of Chinese Society for Environmental Science.

[11] Kulldorff M., Song C.H., Gregorio D., Samociuk H., DeChello L. (2006). Cancer map patterns—Are they random or not?. Amer. J. Prev. Med..

[12] Aragones N., Goicoa T., Pollan M., Militino A.F., Perez-Gomez B., Lopez-Abente G., Ugarte M.D. (2013). Spatio-temporal trends in gastric cancer mortality in Spain: 1975–2008. Cancer Epidemiol..

[13] Belpomme D., Irigaray P., Hardell L., Clapp R., Montagnier L., Epstein S., Sasco A.J. (2007). The multitude and diversity of environmental carcinogens. Environ. Res..

[14] Gao Y., Hu N., Han X.Y., Ding T., Giffen C., Goldstein A.M., Taylor P.R. (2011). Risk factors for esophageal and gastric cancers in Shanxi Province, China: A case–control study. Cancer Epidemiol..

[15] Mayne S.T., Risch H.A., Dubrow R., Chow W.-H., Gammon M.D., Vaughan T.L., Farrow D.C., Schoenberg J.B., Stanford J.L., Ahsan H. (2001). Nutrient intake and risk of subtypes of esophageal and gastric cancer. Cancer Epidemiol. Biomark. Prevent..

[16] Carpenter D.O. (2006). Polychlorinated biphenyls (PCBS): Routes of exposure and effects on human health. Rev. Environ. Health.

[17] Fantini F., Porta D., Fano V., de Felip E., Senofonte O., Abballe A., D’Ilio S., Ingelido A.M., Mataloni F., Narduzzi S. (2012). Epidemiologic studies on the health status of the population living in the Sacco River valley. Epidemiol. Prevenz..

[18] Hendryx M., Conley J., Fedorko E., Luo J., Armistead M. (2012). Permitted water pollution discharges and population cancer and non-cancer mortality: Toxicity weights and upstream discharge effects in U.S. rural-urban areas. Int. J. Health Geogr..

[19] Seker S., Arakawa K., Sekiguchi M., Ono Y. (2005). Biomonitoring of polycyclic aromatic hydrocarbons on hepatocellular carcinoma cell line. Water Sci. Technol..

[20] Wang M., Xu Y., Pan S., Zhang J., Zhong A., Song H., Ling W. (2011). Long-term heavy metal pollution and mortality in a Chinese population: An ecologic study. Biol. Trace Elem. Res..

[21] Zhao H., Guo Q.G., Zhou M.G., Dou Y.S., Yu T., Liu Y.N., Wang X.F., Chen Y.J., Zhang Y.W. (2013). Association between mortality rate of hepatic carcinoma and the distance from Suihe River in Lingbi County, Anhui Province. Chin. J. Prevent. Med..

[22] Tian D., Zheng W., Wei X., Sun X., Liu L., Chen X., Zhang H., Zhou Y., Chen H., Wang X. (2013). Dissolved microcystins in surface and ground waters in regions with high cancer incidence in the Huai River Basin of China. Chemosphere.

[23] Alberto W.D., del Pilar D.A.M., Valeria A.M., Fabiana P.S., Cecilia H.A., de los Ángeles B.M. (2001). Pattern recognition techniques for the evaluation of spatial and temporal variations in water quality. A case study: Suquía River Basin (Córdoba-Argentina). Water Res..

[24] Bengraïne K., Marhaba T.F. (2003). Using principal component analysis to monitor spatial and temporal changes in water quality. J. Hazard. Mater..

[25] Singh K.P., Malik A., Mohan D., Sinha S. (2004). Multivariate statistical techniques for the evaluation of spatial and temporal variations in water quality of Gomti River (India)—A case study. Water Res..

[26] Chiu H.F., Kuo C.H., Tsai S.S., Chen C.C., Wu D.C., Wu T.N., Yang C.Y. (2012). Effect modification by drinking water hardness of the association between nitrate levels and gastric cancer: Evidence from an ecological study. J. Toxicol. Environ. Health. Pt. A.

[27] Wang X.S., Wu D.L., Zhang X.F., Jia S.S., Jin F., Pan Z.Q., Wu M.H., Qin Y. (2009). A case-control study on factors for liver cancer in Ganyu County. Mod. Prevent. Med..

[28] Zhitkovich A. (2011). Chromium in drinking water: Sources, metabolism, and cancer risks. Chem. Res. Toxicol..

[29] Barrett J.H., Parslow R.C., McKinney P.A., Law G.R., Forman D. (1998). Nitrate in drinking water and the incidence of gastric, esophageal, and brain cancer in Yorkshire, England. Cancer Cause. Control.

[30] Nishiwaki-Matsushima R., Nishiwaki S., Ohta T., Yoshizawa S., Suganuma M., Harada K., Watanabe M.F., Fujiki H. (1991). Structure-function relationships of microcystins, liver tumor promoters, in interaction with protein phosphatase. Jpn. J. Cancer Res..

[31] Han C., Jing J.X., Sun G.X. (1997). Study of environmental pollution and damage of cytogenetic materials in urban residents. Chin. J. Epidemiol..

[32] Gallo A., Cha C. (2006). Updates on esophageal and gastric cancers. World J. Gastroenterol..

[33] Guzman R.E., Solter P.F. (2002). Characterization of sublethal microcystin-LR exposure in mice. Vet. Pathol..

[34] Igbinosa E.O., Odjadjare E.E., Chigor V.N., Igbinosa I.H., Emoghene A.O., Ekhaise F.O., Igiehon N.O., Idemudia O.G. (2013). Toxicological profile of chlorophenols and their derivatives in the environment: The public health perspective. Sci. World J..

[35] Xie T.P., Zhao Y.F., Chen L.Q., Zhu Z.J., Hu Y., Yuan Y. (2011). Long-term exposure to sodium nitrite and risk of esophageal carcinoma: A cohort study for 30 years. Dis. Esophagus..

[36] Wu K., Li K. (2007). Association between esophageal cancer and drought in china by using geographic information system. Environ. Int..

[37] Antunes J.L.F., Biazevic M.G.H., de Araujo M.E., Tomita N.E., Chinellato L.E.M., Narvai P.C. (2001). Trends and spatial distribution of oral cancer mortality in São Paulo, Brazil, 1980–1998. Oral Oncol..

[38] Selinus O., Alloway B.J. (2005). Essentials of Medical Geology: Impacts of the Natural Environment on Public Health.

[39] Ministry of Health of the People’s Republic of China (1979). Survey of China’s Deaths from Cancers.

[40] Ministry of Environmental Protection of the People’s Republic of China (2002). Environmental Quality Standards for Surface Water.

