# Supplementary material for: Association between Changing Mortality of Digestive Tract Cancers and Water Pollution: A Case Study in the Huai River Basin, China
Source: Int J Environ Res Public Health. 2014 Dec 23;12(1):214–26. doi: 10.3390/ijerph120100214 (PMC4306858; doi:10.3390/ijerph120100214)
Supplement: Supplementary File 1 [file ijerph-12-00214-s001.pdf]

Supplementary Information

Association between Changing Mortality of Digestive Tract Cancers and Water Pollution: A Case Study in the Huai River Basin, China

Table S1. Linear correlation between CMs and the selected factors at the county scale (with exception of SX County).

	FSP_{WQG}	FSP_{BOD}	FSP_{COD}	FSP_{AN}	DWS
CMT	0.84 ‡	0.82 ‡	0.47	0.87 ‡	−0.58 †
CML	0.74 ‡	0.65 ‡	0.37	0.73 ‡	−0.55 †
CMG	0.77 ‡	0.67 ‡	0.34	0.72 ‡	−0.45
CME	0.60 †	0.56 †	0.29	0.65 ‡	−0.34

Note: †, and ‡ denote the significance level of 0.10, 0.05, and 0.01, respectively (two-tailed).

Table S2. Partial correlation between CMs and FSP indices with various control variables (with exception of SX County).

	Control Variables: GDP-POP-DWS				Control Variables: GDP-POP			
	FSP_{WQG}	FSP_{BOD}	FSP_{COD}	FSP_{AN}	FSP_{WQG}	FSP_{BOD}	FSP_{COD}	FSP_{AN}
CMT	0.71 †	0.63 †	−0.05	0.77 ‡	0.74 ‡	0.68 †	0.08	0.79 ‡
CML	0.53	0.31	−0.18	0.53	0.59 *	0.41	−0.05	0.57 *
CMG	0.67 †	0.43	−0.12	0.56 *	0.65 †	0.48	−0.03	0.59 *
CME	0.36	0.15	−0.15	0.31	0.36	0.19	−0.09	0.33

Note:*, †, and ‡ denote the significance level of 0.10, 0.05, and 0.01, respectively (two-tailed).

© 2014 by the authors; licensee MDPI, Basel, Switzerland. This article is an open access article distributed under the terms and conditions of the Creative Commons Attribution license (http://creativecommons.org/licenses/by/4.0/).
